# User-focused evaluation of National Ecological Observatory Network streamflow estimates

**DOI:** 10.1038/s41597-023-01983-w

**Published:** 2023-02-11

**Authors:** Spencer Rhea, Nicholas Gubbins, Amanda G. DelVecchia, Matthew R. V. Ross, Emily S. Bernhardt

**Affiliations:** 1 Duke University, Durham, NC 27705 USA; 2 Colorado State University, Fort Collins, CO 80523 USA; 3 The University of North Carolina at Chapel Hill, Chapel Hill, NC 27514 USA

**Keywords:** Hydrology, Freshwater ecology, Limnology

## Abstract

Accurately estimating stream discharge is crucial for many ecological, biogeochemical, and hydrologic analyses. As of September 2022, The National Ecological Observatory Network (NEON) provided up to 5 years of continuous discharge estimates at 28 streams across the United States. NEON created rating curves at each site in a Bayesian framework, parameterized using hydraulic controls and manual measurements of discharge. Here we evaluate the reliability of these discharge estimates with three approaches. We (1) compared predicted to observed discharge, (2) compared predicted to observed stage, and (3) calculated the proportion of discharge estimates extrapolated beyond field measurements. We considered 1,523 site-months of continuous streamflow predictions published by NEON. Of these, 39% met our highest quality criteria, 11% fell into an intermediate classification, and 50% of site-months were classified as unreliable. We provided diagnostic metrics and categorical evaluations of continuous discharge and stage estimates by month for each site, enabling users to rapidly query for suitable NEON data.

## Introduction

The flow regime of a river is a fundamental driver of its biology, biogeochemistry, and geomorphology^1^. Streamflow, or stream discharge, influences many ecological processes, including ecosystem respiration^2^, greenhouse gas concentrations and flux^3,4^, and nutrient and element transport^5^. Streamflow is also a fundamental measurement in hydrology used for rainfall-runoff models^6^, fluvial geomorphological applications^7^, and understanding impacts of land use and climate change on hydrologic systems^8^. Streamflow estimates are directly used to estimate gas exchange rates applied in modeling stream metabolism^9,10^ and to calculate greenhouse gas evasion^11^.

Broad scale and continuous estimates of streamflow that correspond to other ecological data are rare. The United States Geological Survey (USGS) collects continuous stream discharge estimates at approximately 13,000 stations nationwide, but gauges are not necessarily collocated with any ecological data, and routine, consistent measurement of any associated variables is generally not a priority^12^. In contrast, many studies have quantified ecosystem processes that are either related to or dependent on direct estimates of streamflow, but at local or temporally limited scales depending on when sampling is possible^13^, which tends to be in warmer months^3,11,14^.

**Fig. 1:**
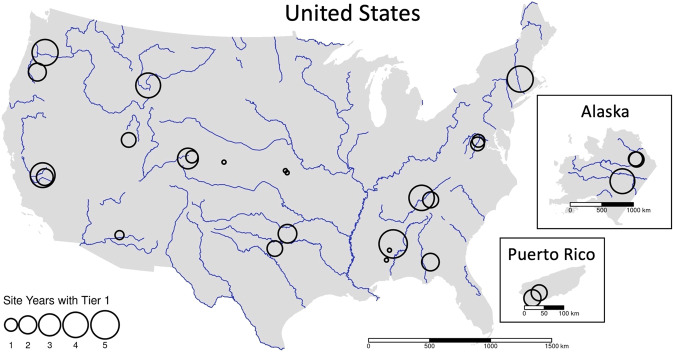
Map of all NEON aquatic sites where NEON estimates streamflow, sized by the total amount of Tier1 data available at the site.

The National Ecological Observatory Network (NEON) provides an unprecedented opportunity to compare up to five years of corresponding, continuously and consistently collected terrestrial, aquatic, and subsurface data from 28 stream and river (referred to as ‘stream’) sites across the United States (Fig. 1). Until 2021, ecological analyses of these data were somewhat limited by a lack of continuous discharge estimates. The NEON Continuous Discharge data product was released in 2021 as DP4.00130.001^15^ and provided both the continuous estimates and associated uncertainty ranges.

Streamflow is traditionally estimated by converting a continuous measurement of water height (stage) to streamflow based on the relationship between stage and discharge^16^. Stage is converted to streamflow using different methods including: empirical equations (such as Manning’s Equation)^17^, empirical rating curves that relate discharge to stage measured across a range of flow conditions^16^, or modeled estimates^18^. One of the most common methods for calculating a time series of streamflow for a particular site is the rating curve method. Although it is widely accepted that rating curves have uncertainty, data is often used as an errorless value in applications^19^. Uncertainty in rating curves can come from many sources including: error in flow measurements^20^, extrapolations beyond measured streamflow^21^, and a poor relationship between stream stage and streamflow.

In order to quantify error in rating curves, NEON used a newer approach that generates streamflow estimates with corresponding uncertainty ranges^22^. This approach, described in detail by Le Coz *et al*., 2017, used a Bayesian framework that combined information from measured streamflow (the likelihood functions) and hydrologic controls (priors) into a “MaxPost” rating curve (the rating curve that relates to the maximum-posterior parameter values)^22^. Using the Bayesian approach provided two benefits over traditional rating curves. One, information on hydrologic controls, such as riffles, large obstructions, and other geomorphic structures which constrain flow could be incorporated into the rating curves^22^. Because different hydrologic features control streamflow at different flow conditions, this method allowed the final rating curve to account for these inflection points. Second, Bayesian methods allowed for quantification of uncertainty associated with rating curves. Having uncertainty associated with a streamflow value is important as many ecohydrologists use streamflow values as absolute although it is known there is almost always uncertainty associated with rating curves^19^. Throughout this paper we will use “predicted streamflow” to refer to the NEON Continuous Discharge dataset.

Manual measurements of streamflow used to construct NEON’s rating curves were collected via acoustic Doppler current profilers (ADCP)^23^, conservative salt tracer releases^24^, and flow meter measurements^25^. It is important to note, each of these methods are used under different conditions and are subject to unique types of error, see citations for details on how the methods are used to calculate streamflow. NEON then used streamflow measurements to construct unique rating curves for each hydrologic year (starting on October 1^st^). Continuous inputs to these rating curve models were generated by converting continuous pressure transducer data to continuous stage (stream surface elevation) using linear regressions with up to 26 manually measured gauge heights per year^15,26^.

To facilitate informed use of NEON streamflow estimates, we evaluated the reliability of discharge estimates for every site-month available by analyzing the quality of continuous stage estimates (rating curve inputs), rating curve fits, and the proportion of streamflow estimates extrapolated beyond field measurements. Using this information, we created a categorical classification set that binned site-months into tiers of reliability, and flagged data that was of lesser quality. Although NEON’s Bayesian approach to modeling discharge reports uncertainty, the Continuous Discharge product contained sixteen columns that related to various sources of uncertainty and nine columns of quality control flags in the expanded data product, making using this information effectively complicated.

Our evaluation dataset includes categorical classifications of NEON’s Continuous Discharge product that allows users to easily query tabular data for reliable discharge estimates by month at all 28 sites where NEON provides estimates of streamflow using commonly used metrics like Nash-Sutcliffe coefficients. The classification dataset includes flags for potential sensor drift at sites (“drift flag”), flags for weak relationships between stage and gauge height (“regression flag”), and a final three tier rating for the reliability of streamflow estimates. “Tier 1” corresponds to the data that is ready to use for most hydrologic analyses, “Tier 2” indicates data that may be unreliable at high streamflow values or has a weaker fit of stage and streamflow overall, and “Tier 3” identifies data that is likely unreliable and should only be used after close inspection. We hope this dataset can increase the informed use of NEON streamflow estimates for ecological questions at a continental scale.

## Results

To facilitate use of the NEON Continuous Discharge dataset, we evaluated and classified site-months into various categories to inform users on potential uncertainty in stage measurements and rating curve fits. Based on the availability of NEON discharge data, we were able to consider 1,523 site-months of continuous streamflow. Our evaluation included multiple tests to determine the quality of streamflow data. The first part of our evaluation considered the quality of the continuous stage datasets which is used as the inputs for NEON’s rating curves. NEON predicted continuous stage by converting continuous in-stream pressure transducer readings to water height using linear regressions parameterized with field measurements of water height. We calculated the Nash-Sutcliffe model efficiency coefficients (NSE) for these regressions and tested if continuous pressure transducer measurements of stage deviated from manual measurements of gauge height. Next, we evaluated the fit of rating curves used to convert this continuous stage to continuous discharge, by calculating a NSE for each model and the proportion of reported continuous discharge values extrapolated beyond field measurements.

### Stage classification

**Fig. 2:**
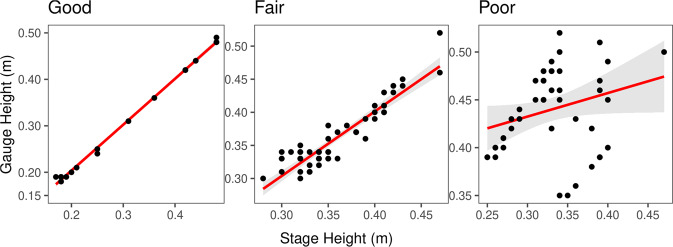
Examples of NEON stage-gauge height regression falling into each of our classifications: Teakettle Creek (TECR) 2019 relationship (good), Lewis Run (LEWI) 2017-1 relationship (fair), and LeConte Creek (LECO) 2017-2 relationship (poor).

Regression classification found 56% of site months with good regression (NSE > 0.9) between continuous stage and gauge height, 8% with fair regressions (0.75 < NSE < 0.90), 31% with poor regressions (NSE < 0.75), and 5% were not classified due to inadequate data. Figure 2 shows an example of a regression relationship falling into each of the three categories.

### Drift detection

**Fig. 3:**
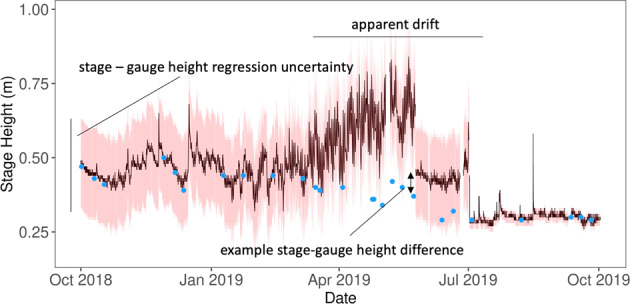
Continuous gauge height at the Lewis Run (LEWI) site, continuous gauge height (black), uncertainty (red), and manual stage height (blue). Pressure transducers are subject to occasional drift, here we use manual measurements of stage to ensure pressure data is consistent with real stream stage. Uncertainty bounds are produced by NEON and are available in the Elevation of Surface Water product.

Comparing manual measurements of stage height to continuous gauge height and uncertainty ranges, allowed us to determine periods of potential sensor drift (Fig. 3). Drift detections found 57% of site-months likely had no drift, 8% of sites had potential drift, and the remaining 35% of site-months could not be assessed for drift because there were no manual measurements of stage height. If a site could not be assessed, it did not affect a sites-month’s classification but the inability to asses drift over the month is noted in the evaluation dataset.

### Rating curve classification

**Fig. 4:**
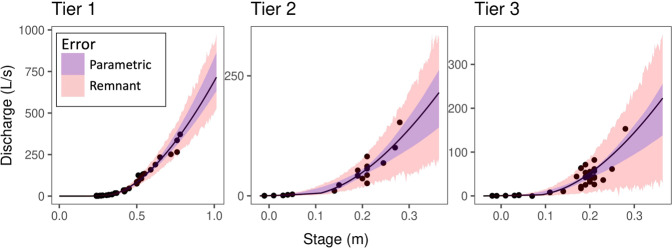
Examples of NEON rating curves falling into each of our classifications: Como Creek (COMO) 2018 curve (Tier 1, NSE = 0.97), Arikaree River (ARIK) 2016 curve (Tier 2, NSE = 0.87), and ARIK 2017 curve (Tier 3, NSE = 0.74). Parametric error in purple and remnant error in red produced by NEON.

Rating curves were classified based on the efficiency of the rating curves (NSE) and the amount of continuous discharge values extrapolated beyond field measurements. Rating curve classification found 67% of site-months with Tier 1 rating curves, 19% with Tier 2 rating curves, and 17% with Tier 3 rating curves (see Table 1 and methods for description of the three tiers). Figure 4 shows an example of rating curves falling into each of the three tiers.

**Table 1:** Rating curve classification criteria.

Classification	Nash-Sutcliffe Coefficient	Extrapolated beyond maximum gauging
Tier 1	NSE > 0.9	<15%
Tier 2	0.75 < NSE < 0.9	<30%
Tier 3	NSC < 0.75	>30%

**Fig. 5:**
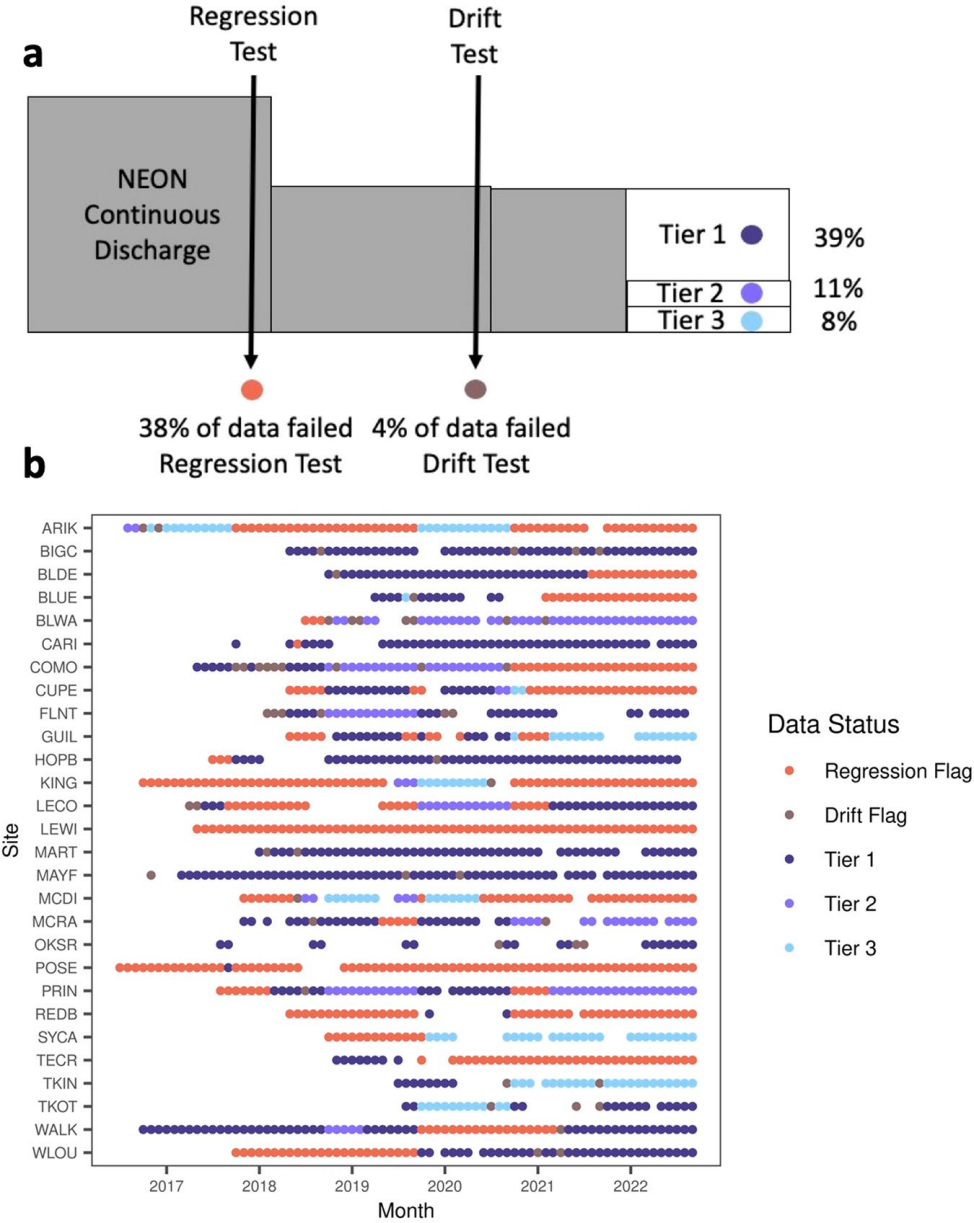
Panel a, conceptual diagram depicting how data is partitioned into final evaluation category with the percentage of site-months with each flag or classification. Panel b, NEON monthly site classification for stage-gauge regressions, drift, and rating curves. Points colored by rating curve classification (Tier 1, 2, 3) if the regression status is good and no drift was detected in that month, otherwise colored red or brown to indicate flags. Missing points indicate no data is available for that month.

To determine the final classification of a site-month’s quality, our workflow first asked if the input data to the rating curve was reliable. If the relationship between the stage and gauge height was unreliable (a fair or poor classification), we report a regression flag. Second, we checked if a site-month had potential drift. If the data did have potential drift, we reported a drift flag. Finally, if these tests found the input stage data were reliable and there was no detectable drift, then the rating curve classification is reported as the final quality assessment (Fig. 5, Panel A).

Of the 1,523 site-months of continuous streamflow predictions published by NEON, 38% were classified in our highest quality category of Tier 1. 11 of the 28 NEON sites had at least one full water year of Tier 1 streamflow data. Of all data classified in our analysis, site-months with unreliable inputs to the discharge models (predicted stage values) comprised the largest proportion of site-month classifications, with 38% of site months resulting in a regression flag (Fig. 5).

Figure 5 outlines how data moves through our classification scheme and the final results for each site-month.

## Discussion

Our goal with this formal evaluation of the NEON Continuous Discharge product was to enable researchers to rapidly identify high quality data that could be used in subsequent analyses and to reduce the likelihood that consumers of this dataset invest effort in conducting analyses with unreliable data. Streamflow data that was classified as Tier 1 met the high professional standards of the hydrology community and can be used in all subsequent analysis with confidence. This accounted for 39% of the data released by NEON. More than half of the data provided by NEON should be used with caution.

About 11% of the published NEON streamflow data were classified as Tier 2 which means that more than 15% of reported values are beyond the maximum manual measurement of streamflow, or that the fit between the rating curve and stage measurements was weaker than for Tier 1 (Nash Sutcliffe coefficients 0.75–0.9). For data in this tier, low to intermediate flow data is likely reliable, but users are discouraged from calculating annual fluxes of solutes or gases from data where stormflows may be significantly over or underestimated, or modeling metabolism or hydrologic exchange during periods of higher flow. These cautions are repeated and made stronger for the ~8% of the dataset that was classified as Tier 3 because more than 30% of their reported streamflow values were beyond the maximum manual estimate of discharge or because the fit between the rating curve and stage measurements were poor (Nash Sutcliffe <0.75).

Nearly half (42% of the data) were not ranked into one of these three tiers because of poor fits between stage and gauge height measurements that caused us to question the quality of the data. There are a variety of reasons that might lead to the large uncertainty around both readings (e.g., field error, sensor failure, movement of sensors or gauges without record, channel reshaping following floods) but we have insufficient information with which to identify or correct for any of these potential errors. We have imposed Tier rankings in our evaluation, but the quality of the NEON discharge product spans a continuous gradient. Users are encouraged to set their own criteria for use, and all of the evaluative metrics are available to assist them in doing so.

Although we determined that a large proportion of the streamflow data currently provided by NEON are unreliable, some sites provide more than a year of reliable data that can be used, and the extent of reliable data has been increasing over time. We urge NEON site managers and field crews to devote increased attention to collecting manual discharge estimates at high and low flows to improve their rating curves and increase the availability of highly reliable data in the future. We encourage all prospective users of NEON discharge data to engage in efforts to improve upon our current effort. For example, efforts to estimate discharge in ungauged basins may prove useful in supplementing and refining the unreliable data collected for the early years of NEON. Improvements in the NEON Continuous Discharge product will offer many exciting opportunities to combine an unprecedented set of hydrologic, biogeochemical, biological, and ecological data from streams across the United States. Our intention with this paper is to enable those exciting analyses to occur.

## Methods

As part of the streamflow data release, NEON released four relevant data products: Gauge Height^26^, Elevation of Surface Water^29^, Stage-discharge Rating Curves^30^, and Continuous Discharge^15^. Data users are able to download this full suite of information and protocols to inform decisions on data usage and applicability. We evaluated the quality of the Continuous Discharge product using all four relevant NEON data products, considering the validity of model inputs as well as the goodness-of-fit of final streamflow estimates. We analyzed 1) the fit of the regression between manual stage height readings and continuous pressure transducer data used to estimate continuous stream surface elevation, 2) the fit of rating curves transforming stream surface elevation to streamflow, and 3) the proportion of streamflow estimates over the maximum manually-measured streamflow.

### Stage classification

The rating curve models predicting streamflow required continuous stream stage estimates as model inputs. NEON predicted continuous gauge height with a two step approach. First, continuous in-stream transducer readings were converted to water height by applying an offset between the transducer elevation and the staff gauge (Eq. 1). This offset is derived from the NEON geolocation database as the difference between the location of the pressure transducer and the staff gauge^27^. The offset changes only when the location of either the staff gauge or transducer moves.

1 $${h}_{wc}=\frac{{P}_{sw}}{p\,\ast \,g}\,\ast \,1000+{h}_{stage}$$

Conversion of pressure data to water height used by NEON^27^ where *h*_*wc*_ is the estimated water column height (m), *P*_*sw*_ is calibrated surface water pressure (kPa), p is the density of water (999 kg/*m*^3^), g is the acceleration due to gravity (9.81 *m/s*^2^), and *h*_*stage*_ is the offset between the pressure transducer and the staff gauge (m).

Then, NEON uses a linear regression between manually-measured reference stage height and the calculated gauge height from Eq. 1, yielding final predictions of continuous stream gauge height^27^. In an ideal setting, stage and gauge height should correlate perfectly^28^. In the field, sensor uncertainty, manual reference measurement error, and shifting conditions in the stream can convolute the relationship. We tested the goodness of fit between continuously estimated stream gauge height values and manual stage measurements using the Nash-Sutcliffe model efficiency coefficient (Eq. 2). Nash-Sutcliffe coefficient is a commonly used metric in hydrology used to evaluate how well a model performed relative to observed values (manually measured stage and calculated gauge height). For the purposes of this discussion, manual reference measurements will be referred to as ‘stage’ and automated, sensed readings as ‘gauge height’.

2 $$NSE=1-\frac{\Sigma {\left({Q}_{o}-{Q}_{m}\right)}^{2}}{\Sigma {\left({Q}_{o}-{\bar{Q}}_{o}\right)}^{2}}$$

Equation 2 presents Nash-Sutcliffe model efficiency coefficient, where *Q*_*o*_ is an observed value (streamflow or stage height), *Q*_*m*_ is a modeled value, and ${\bar{Q}}_{o}$ is the mean of observed values.

Stage, gauge height, and regression data were sourced from the NEON Continuous Discharge product, representing what was directly applied to streamflow estimation. Up to 26 stage measurements were available per year. We examined every regression between stage and gauge height (one per site year in which data was available) and classified each as either ‘good’, ‘fair’, or ‘poor’ quality based on their goodness of fit. Regressions with a NSE (Eq. 2) of 0.90 or greater were considered good, those with a NSE of less than 0.90 but greater than or equal to 0.75 were considered fair, and those with an NSE of less than 0.75 were considered poor (Fig. 2).

### Drift detection

Because electronic instruments, such as pressure transducers, can have systematic directional drift, referred to as ‘drift’, during deployment, we developed an approach to detect periods of time when NEON’s Elevation of Surface Water product drifted. We used two methods to assess and flag the potential for instrument drift at monthly time steps. First, we flagged any period the manually measured stage fell outside NEON’s uncertainty bound for gauge height made at the same time. From this, we calculated the proportion of stage measurements outside of the gauge height uncertainty bounds per month. This proved to be a relatively lenient filter that missed periods of manually identified drift. We found adding a second filter that flagged any month where the difference between the manually measured stage and gauge height exceeded 6 cm, was effective in catching the majority of periods where drift was identified. Second, we calculated the average differences between stage and gauge height for each month (Fig. 3). To determine appropriate cut-off values to classify areas of potential drift, we manually audited and flagged periods of observable directional drift. Our goal was to set a maximum cut-off difference which retained as much usable data as possible while still capturing 70% of the manually flagged directional drift periods. Applying this method, we determined a cut-off value of 6 cm average monthly deviation between observed and predicted stage values.

Using these two filters in combination, we again classified data into three groups: ‘likely no drift’, ‘potential drift’, and ‘not assessed’. Site-months with no more than 50% of stage measurements outside of the gauge height time series uncertainty and an average difference between stage and gauge height less than 6 cm were considered to have ‘likely no drift’. Site-months with either more than 50% of stage readings outside of the gauge height time series uncertainty or an average difference between stage and gauge height more than 6 cm were deemed to have ‘potential drift’. Site-months with no stage measurements could not be evaluated and were considered ‘not assessed’. Although this approach to identify drift is imperfect, in that slight drift could be missed and times without manual measurements are not possible to assess, we believe this is a helpful method given the data available from NEON and the fact drift has been observed when visually inspecting data (Fig. 3).

### Rating curve classification

To evaluate how well rating curves predicted streamflow, we assessed each rating curve used to convert stage to discharge. NEON prepares a new rating curve for each site’s water year (beginning on October 1st)^27^. In cases where NEON reported multiple rating curves for a site’s water year each curve was assessed separately across the time series which it was used. We classified rating curves into three tiers based on two metrics: the Nash-Sutcliffe coefficient (Eq. 2) between observed and predicted streamflow, and the percentage of continuous discharge values above the maximum manually measured gauging used to construct the rating curve.

First, we calculated the Nash-Sutcliffe coefficient for each rating curve to estimate how well rating curves captured the variation in the stage-streamflow relationship. We used the reported values for modeled and manually measured streamflow from the ‘Y1simulated’ and ‘Y1observed’ columns in the ‘sdrc_resultsResiduals’ table of the Stage-discharge rating curves product. NEON generally conducts between 12 and 24 manual gaugings per year to build and maintain the stage-discharge relationship.

Second, we calculated the percentage of continuous streamflow values outside the range of manually measured estimates of streamflow. This was useful to assess if the stage-discharge relationship is representative of observed flow conditions. The relationship between discharge and stage is often nonlinear, with inflection points around changes in channel morphology making gauging the stream at high and low flow conditions critical to building a reliable rating curve^16^. A rating curve based on a large number of direct field measurements all taken during a narrow range of baseflows, for example, could generate a rating curve with a high Nash-Sutcliffe coefficient that is unreliable when extrapolated to high or low flow events. Using these two metrics, we were able to classify rating curves into categories of relative quality. To calculate the percentage of values in the continuous streamflow product that fall outside the range of manually gauged streamflow values, we extracted the maximum and minimum gauging values from the ‘sdrc_resultsResiduals’ table in the Stage-discharge Rating Curve product. We then compared the predicted values derived from each rating curve (as reported in the ‘csd_continuousDischarge’ table) to the extracted range and calculated the proportion of values which fell outside of it.

We used the Nash-Sutcliffe coefficient and percentage of streamflow values over the maximum observed field measurements to classify rating curves into three categories outlined in Table 1.

To integrate stage-gauge regressions, drift detections, and rating curve classification, we produced a summary table with classifications for all three tests and the corresponding metrics used in each classification (Fig. 5). The table is grouped by month and site so users can query sites and determine which months have the appropriate data for their needs.

## Data Availability

The results of this analysis are available at the cited HydroShare repository^31^. All data evaluated in this publication are collected by the National Ecological Observation Network and made available through NEON’s web portal. Data was accessed using the loadByProduct function in the R Package neonUtilities^32^. We evaluated the continuous discharge product^15^ and Stage-discharge rating curve product^30^ for use in various stream ecology analyses. The RELEASE-2022 portions of the evaluation datasets are available at the cited refs. ^15,30^. The PROVISIONAL portions of the evaluation datasets were accessed on 10/12/2022 from https://data.neonscience.org/data-products/DP4.00130.001 and https://data.neonscience.org/data-products/DP4.00133.001, however these are no longer available as per NEON policy.

## Code availability

All code used in this analysis is available in the cited github repository^33^. This has been annotated to make it easy for users to see what portion of the NEON stream discharge data product is being used and to easily alter the code to analyze subsets of the data specific to their intended use. Code was run using the neonUtilities R package version 2.1.2^32^ and R version 4.1.2^34^.
