# Author Correction: User-focused evaluation of National Ecological Observatory Network streamflow estimates

**DOI:** 10.1038/s41597-023-02026-0

**Published:** 2023-02-23

**Authors:** Spencer Rhea, Nicholas Gubbins, Amanda G. DelVecchia, Matthew R. V. Ross, Emily S. Bernhardt

**Affiliations:** 1 Duke University, Durham, NC 27705 USA; 2 Colorado State University, Fort Collins, CO 80523 USA; 3 The University of North Carolina at Chapel Hill, Chapel Hill, NC 27514 USA

**Keywords:** Hydrology, Freshwater ecology, Limnology

Correction to: *Scientific Data* **10**:89; 10.1038/s41597-023-01983-w, published online 11 February 2023.

In this article the author name Matthew R.V. Ross was incorrectly written as Matthew R. Ross. The original article has been corrected.

