# Author Correction: User-focused evaluation of National Ecological Observatory Network streamflow estimates

**DOI:** 10.1038/s41597-023-02671-5

**Published:** 2023-10-31

**Authors:** Spencer Rhea, Nicholas Gubbins, Amanda G. DelVecchia, Matthew R. V. Ross, Emily S. Bernhardt

**Affiliations:** 1 Duke University, Durham, NC 27705 USA; 2 Colorado State University, Fort Collins, CO 80523 USA; 3 The University of North Carolina at Chapel Hill, Chapel Hill, NC 27514 USA

**Keywords:** Hydrology, Freshwater ecology, Limnology

Correction to: *Scientific Data* 10.1038/s41597-023-01983-w, published online 11 February 2023

The Data Availability Statement was incomplete in the original version, failing to acknowledge that both the PROVISIONAL and RELEASE-2022 data products for the continuous discharge product and stage-discharge rating curve product were downloaded and analysed from NEON (National Ecological Observatory Network). While the RELEASE-2022 datasets were cited in the original work (references 15 and 30), no information was present to explain how and when the provisional data were obtained. The following text has been added to the Data Availability Statement to clarify this, in the pdf and HTML versions of the article:

“The RELEASE-2022 portions of the evaluation datasets are available at the cited references.15,30 The PROVISIONAL portions of the evaluation datasets were accessed on 10/12/2022 from https://data.neonscience.org/data-products/DP4.00130.001 and https://data.neonscience.org/data-products/DP4.00133.001, however these are no longer available as per NEON policy.”

To provide additional clarity, the code associated with the analysis has been updated to make it easier for users to see what portion of the NEON stream discharge data product were used and to make it easy for users to alter the code to analyze subsets of the data specific to their intended use. This information has been added to the Code Availability statement in the pdf and HTML versions of the article.

